# Oxidative stress generated during monensin treatment contributes to altered *Toxoplasma gondii* mitochondrial function

**DOI:** 10.1038/srep22997

**Published:** 2016-03-15

**Authors:** Robert A. Charvat, Gustavo Arrizabalaga

**Affiliations:** 1 Departments of Pharmacology and Toxicology Indiana University School of Medicine, Indianapolis, Indiana 46202, US; 2 Departments of Microbiology and Immunology, Indiana University School of Medicine, Indianapolis, Indiana 46202, US.

## Abstract

The ionophore monensin displays potent activities against several coccidian parasites of veterinary and medical importance including the opportunistic pathogen of humans, *Toxoplasma gondii*. While monensin is used widely in animals, toxicity impedes its use in humans. Nonetheless, given its potency, understanding its mode of action would reveal vulnerable aspects of the parasite that can be exploited for drug development. We previously established that monensin induces *Toxoplasma* to undergo cell cycle arrest and an autophagy-like cell death. Interestingly, these effects are dependent on the mitochondrion-localized TgMSH-1 protein, suggesting that monensin disrupts mitochondrial function. We demonstrate that monensin treatment results in decreased mitochondrial membrane potential and altered morphology. These effects are mitigated by the antioxidant compound N-acetyl-cysteine suggesting that monensin causes an oxidative stress, which was indeed the case based on direct detection of reactive oxygen species. Moreover, over-expression of the antioxidant proteins glutaredoxin and peroxiredoxin 2 protect *Toxoplasma* from the deleterious effects of monensin. Thus, our studies show that the effects of monensin on *Toxoplasma* are due to a disruption of mitochondrial function caused by the induction of an oxidative stress and implicate parasite redox biology as a viable target for the development of drugs against *Toxoplasma* and related pathogenic parasites.

The apicomplexan parasite *Toxoplasma gondii* is capable of infecting virtually any nucleated cell from homeothermic animals and resides in approximately one-third of the world’s human population. Transmission of *T. gondii* can occur via ingestion of infectious oocysts shed by the definitive feline host that contaminate water and vegetation^1^, consumption of undercooked meat from chronically infected animals^2^, vertical transmission^3^, and even through blood and tissue products used for transplants^4^,^5^. Due to the multiple routes of infection, its high prevalence, and world-wide distribution, this protozoan pathogen has been aptly given the auspicious moniker of one of the most successful parasites on earth. While infection in immunocompetent individuals is generally asymptomatic and self-limiting, primary infection or reactivation of latent infection in immunocompromised individuals, such as those undergoing cancer chemotherapy, immunosuppression for organ transplantation, or AIDS patients, can be fatal^6^. Current chemotherapeutic regimens involve the drugs pyrimethamine and sulfadiazine^7^, which are efficient at treating acute toxoplasmosis, albeit with potential toxic side effects; but they are disappointingly ineffective against the encysted bradyzoite form that is responsible for establishing a chronic infection. Thus, an imperative need exists for discovering and developing novel therapies against *T. gondii*.

The polyether ionophorous antibiotic monensin was previously demonstrated to be efficient at killing *in vitro* and *in vivo* developed *T. gondii* bradyzoite cysts as well as inhibiting the shedding of infectious oocysts from experimentally infected felines^8^,^9^. Nonetheless, while this effective drug is utilized in poultry and ruminants to prevent infections with related coccidian parasites such as *Eimeria spp.* and *Cryptosporidium spp.*^10^,^11^,^12^, it is not routinely used in human patients due to concerns with toxicity^13^,^14^. Nevertheless, given its high potency, determining the mechanism of action would provide valuable insights for the development of novel therapeutics or repurposing current therapeutics that possess similar functional properties. To this end, we have focused on characterizing the effects of monensin against *T. gondii* and have previously reported that treated parasites undergo a disruption of cell cycle progression that precedes events that resemble an autophagy-like death process^15^,^16^. Specifically, we observed that prolonged monensin treatment resulted in the autophagy related protein TgATG8 being relocalized from a diffuse cytoplasmic pattern to a concentrated punctate pattern reminiscent of autophagosomes. Furthermore, addition of the autophagy inhibitor 3-methyladenine prevented TgATG8 relocalization and protected the parasite against monensin-induced death. Interestingly, it was noted that during treatment with monensin, the parasite mitochondrion lost its characteristic annular appearance^17^ and appeared as discontinuous puncta, suggesting breakdown of the overall mitochondrial architecture. Remarkably, at the same time point after treatment the nucleus, apicoplast, and plant-like vacuole remain mostly unaltered^16^. Further pointing at the mitochondrion as a possible target for monensin action was the fact that disruption of a mitochondrion-localized MutS homolog (TgMSH-1) resulted in resistance to monensin^18^. In conjunction, these results have led us to develop a model for monensin action in which monensin directly or indirectly affects the mitochondrion resulting in the induction of the cell cycle disruption and an autophagy-like process through a signaling pathway that depends on TgMSH-1. While we have learned about the mechanisms involved in monensin-induced death, we still don’t know the cellular effects that lead to this death process and whether the mitochondrion of the parasite is directly involved.

The mitochondrion is the cellular powerhouse, generating energy in the form of ATP through oxidative phosphorylation. Disruption of mitochondrial function can have devastating consequences for a cell and, not surprisingly, is often the goal of many anti-microbial agents. In the case of *Toxoplasma* and the closely related malaria parasite *Plasmodium falciparum*, the cytochrome *b* inhibitor atovaquone is often used to combat human infections with either of these pathogenic parasites^19^,^20^. A variety of other anti-parasitic compounds have been shown to target the mitochondrion, including azasterols^21^, bisphosphonates^22^, and 8-hydroxyquinoline^23^. Focusing on the parasite mitochondrion as an anti-parasitic target has been a priority in the drug development field for decades^24^,^25^,^26^. Key to efficient mitochondrial function is the electrochemical gradient across the inner mitochondrial membrane (IMM). As a Na^+^/H^+^ exchanging ionophore^27^, monensin is expected to disrupt ionic gradients across cellular membranes^28^. In various rat tissues including testis, liver, and lung, monensin was demonstrated to interfere with the proper shuttling of protons across the IMM leading to a loss in mitochondrial membrane potential as well as interruption of the electron transport chain, oxidative phosphorylation, and ATP production^29^,^30^. In murine fibroblasts, monensin-induced toxicity was shown to create an ionic imbalance that greatly affected the morphology of mitochondria^14^,^31^. Disruption of mitochondrial function results in the generation of reactive oxygen species; and indeed, monensin was demonstrated to be a potent inducer of oxidative stress in prostate cancer cells^32^. Considering our previous observations that suggested the mitochondrion was the functional target of monensin, determining whether monensin elicits specific alterations in mitochondrial function in *T. gondii* was a critical step in elucidating the mode of action of this potent drug.

In the present study, we report that monensin indeed directly affects mitochondrial function and is a potent inducer of sustained oxidative stress. Importantly, we show that over-expression of the antioxidant proteins glutaredoxin and peroxiredoxin 2 offered protection against the monensin-induced effects on the mitochondrion. The data presented here offers a detailed assessment of the mechanism of action for monensin, whereby the drug causes an oxidative stress and severely compromises the integrity of the sole mitochondrion of *T. gondii* leading to parasite death. This knowledge provides a rationale for developing redox active drugs or repurposing current chemotherapeutics with similar functional properties for the effective treatment of toxoplasmosis, and by extension other parasitic infections.

## Results

### Monensin disrupts *T. gondii* mitochondrial membrane potential

**Figure 1:**
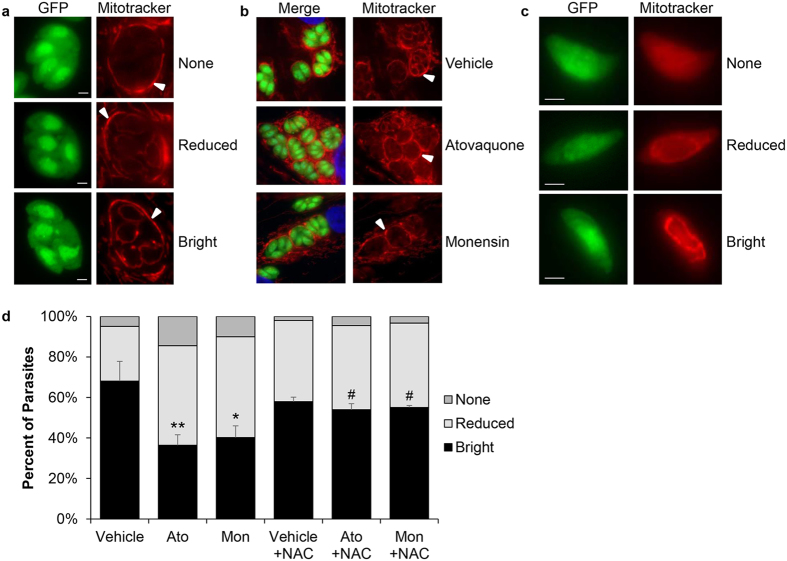
Monensin treatment reduces MitoTracker staining, which is reversed by the addition of NAC. Human foreskin fibroblasts (HFF) were infected with GFP-expressing *Toxoplasma* parasites and treated for 5 hours with vehicle, atovaquone (Ato, 10 nM), or monensin (Mon, 1 ng/mL) in the presence or absence of N-acetyl-cysteine (NAC, 50 μM) then stained intracellularly (**a**,**b**) or extracellularly (**c**) with 200 nM MitoTracker Red CMXRos. (**a**,**b**) MitoTracker stains parasites residing within the parasitophorous vacuole as well as the host cell mitochondria (white arrowheads). (**d**) Staining patterns were enumerated for 100 extracellularly stained parasites from each treatment condition and presented as percent ± SD. Data represents 3 independent experiments and statistical analysis was performed using student’s t-test, where **p < 0.003, *p < 0.026, and ^#^p < 0.017. Scale bars equal 2 μm.

Considering our discovery that genetic disruption of a mitochondrion protein resulted in resistance to the anti-parasitic drug monensin, we investigated the effects of monensin treatment on *T. gondii* mitochondrial biology to gain insights into a potential mechanism of action. In order for the mitochondrion to complete oxidative phosphorylation and generate ATP, an intact electrochemical gradient across the inner mitochondrial membrane (IMM) must be established and maintained. This gradient, also known as the mitochondrial membrane potential (ΔΨm), can be assessed using cationic fluorescent dyes that accumulate in the mitochondrial matrix so long as the ΔΨm remains intact^33^,^34^. Utilizing the red fluorescent probe MitoTracker^35^,^36^, we explored whether monensin treatment alters the *T. gondii* mitochondrial membrane potential. For this purpose, intracellular parasites were treated for 5 hours with monensin; vehicle, as a negative control; or, atovaquone, which has been reported to disrupt ΔΨm^20^, as a positive control. Following treatment, cultures were stained with MitoTracker, and intracellular parasites were examined for changes in membrane potential as indicated by decreased red fluorescence (Fig. 1a). It is important to note that MitoTracker stains all intact mitochondria including those of the host cell, which can be clearly observed surrounding the parasitophorous vacuole (Fig. 1a,b, white arrowheads). Qualitatively, three patterns of mitochondrial staining within the parasites were observed: while the vast majority of vehicle treated parasites possessed a mitochondrion fluorescing brightly, it was noted that a portion (generally less than 30%) of vehicle treated parasites consistently displayed reduced or no MitoTracker staining, a phenomenon observed previously in normally growing parasites^37^. Examining drug-treated parasites, it was noted that, as expected, atovaquone resulted in fewer vacuoles containing brightly stained parasite mitochondria when compared to vehicle treatment (Fig. 1b). Likewise, monensin treatment yielded decreased numbers of vacuoles with brightly stained mitochondria, suggesting that monensin disrupts maintenance of mitochondrial potential in dividing tachyzoites (Fig. 1b). None of these treatments affected the brightness of host mitochondria, which is not surprising since we are using concentrations at least 50 times below those used to elicit changes in mammalian host mitochondria^13^,^29^,^31^.

**Figure 2:**
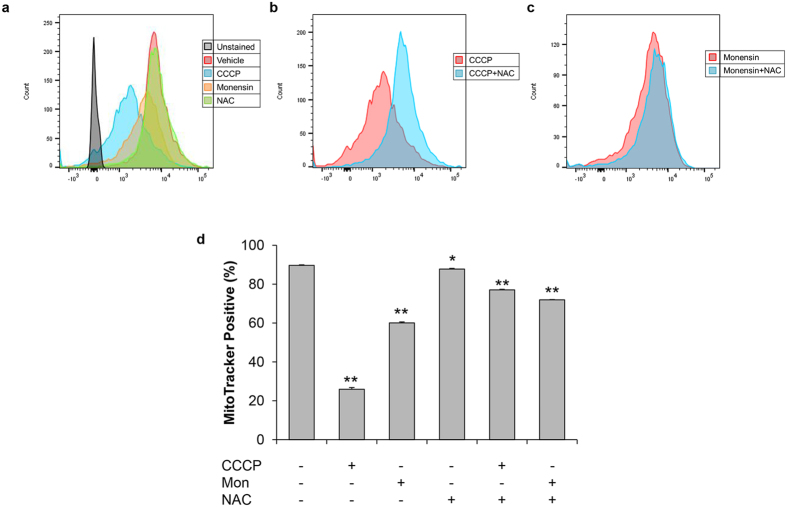
NAC protects against membrane potential disruption caused by CCCP and monensin. Flow cytometric analysis of MitoTracker staining in parasites treated with vehicle, CCCP (1 μM), or monensin (1 ng/mL) for 5 hours in the presence or absence of NAC (50 μM). (**a–c**) Representative distribution of parasites grown in the specified treatments. (**d**) The percentage of MitoTracker positive parasites by flow cytometry was quantified from 3 independent experiments. Error bars represent standard deviation. Statistical analysis was provided by student’s t-test, where **p < 0.001 and *p < 0.02.

Due to the high background MitoTracker staining in the host cells (Fig. 1b), quantitative analysis of MitoTracker staining in the parasites proved challenging. To overcome this, parasites were stained extracellularly following intracellular drug treatment and manual release. We observed the same staining patterns in the extracellularly stained parasites as with the intracellular ones, those being bright, reduced, and no MitoTracker stain (Fig. 1c). It should be noted that under these treatment conditions, the mitochondrion remains intact as exemplified by the characteristic annular morphology for this organelle^17^. As seen in intracellular parasites, monensin treatment reduced the proportion of brightly stained mitochondria from 69.25% ± 9.95% in vehicle treated parasites to 41.25% ± 5.85% (Fig. 1d). This reduction in the proportion of parasites with bright MitoTracker stain is similar to what was detected with the positive control drug atovaquone (reduced to 36.5% ± 5.2%). Furthermore, the proportion of parasites with no staining, which could be unequivocally scored without need for qualitative assessment, revealed that the percentage increased from 5% ± 2.45% in vehicle treated parasites to 10.25% ± 5.97% in monensin treated parasites. These results suggest that monensin is capable of disrupting mitochondrial membrane potential. To corroborate our microscopic assessment of MitoTracker staining, we utilized flow cytometric analysis to detect shifts in fluorescence following monensin treatment^38^,^39^. After 45 minutes of staining, there was a definitive accumulation of stain within the parasites as compared to unstained control cells (Fig. 2a). The mitochondrial uncoupler CCCP was used as a positive control to demonstrate collapsed ΔΨm, which is indicated by a clear decrease in MitoTracker staining (Fig. 2a). CCCP treatment reduced the percentage of parasites positive for MitoTracker staining from 89.73% ± 0.23% in vehicle treatment to 25.97% ± 0.91% (Fig. 2d). Following monensin treatment, we again observed reduced MitoTracker staining compared to vehicle treated parasites, indicated by the leftward shift in fluorescence (Fig. 2a). Though the decrease in MitoTracker positive parasites was not as great as with CCCP, the reduction to 60.07% ± 0.55% was still statistically significant (Fig. 2d). Altogether, we conclude that monensin is capable of reducing mitochondrial membrane potential in *T. gondii*.

### N-acetyl-cysteine protects against drug-induced ΔΨm disruption

Disruption of the mitochondrial membrane potential can indicate organelle dysfunction. Studies in various mammalian cells have demonstrated that initial reactive oxygen species (ROS) generation can result in depolarization of the inner mitochondrial membrane, collapse of the intact membrane potential, and a subsequent “burst” of additional ROS, a phenomenon known as ROS-induced ROS release^40^,^41^,^42^,^43^. To determine whether reactive oxygen species are involved in the drug-induced loss of membrane potential, parasites were co-treated with drug and N-acetyl-cysteine (NAC), a ROS scavenger. Again, intracellular parasites were treated for 5 hours, then manually released and stained extracellularly with MitoTracker. In the presence of NAC, the proportion of parasites with brightly stained mitochondria was reduced by neither atovaquone (54% ± 2.94%) nor monensin (55.25% ± 0.96%) as compared to the untreated control 58.25% ± 2.22% (Fig. 1d). Moreover, the addition of NAC to the monensin treated parasites reduced the proportion of parasites with no MitoTracker staining from 10.25% ± 5.97% down to 3.25% ± 2.75%. Again we utilized flow cytometry to corroborate our microscopic evaluation, which demonstrated that NAC addition to monensin-treated parasites offered some protection against the disruption in Δψm, increasing the percentage of MitoTracker positive parasites from 60.07% ± 0.55% in monensin treatment to 72.00% ± 0.10% in monensin plus NAC treatment (Fig. 2c,d). Likewise, addition of NAC to CCCP treatment also protected against Δψm disruption, increasing the proportion of MitoTracker positive parasites by 51% (Fig. 2b,d). To ensure the effect was not specific to MitoTracker, we used additional membrane potential-sensitive dyes, JC-1^44^ and TMRE^45^, and flow cytometry to further validate our observations. The results from the JC-1 and TMRE experiments mirrored those of the MitoTracker experiments (Supplementary Fig. S1). Altogether, these results indicate that scavenging reactive oxygen species can protect against some of the deleterious effects of monensin, suggesting that an oxidative stress may be involved in the mechanism of action for monensin.

### Scavenging reactive oxygen species during monensin treatment partially protects against mitochondrial remodeling

**Figure 3:**
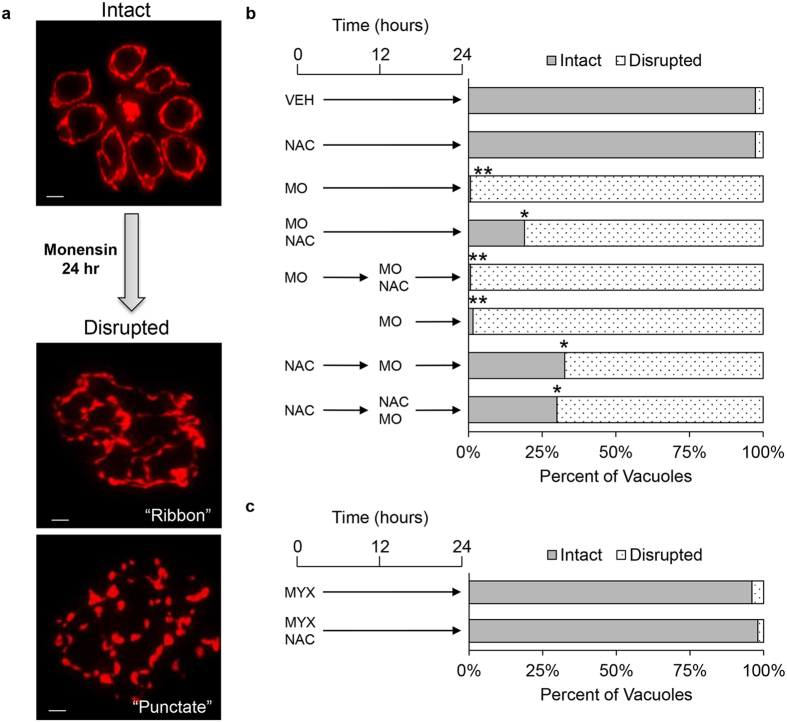
Monensin disrupts parasite mitochondrial morphology, which is partially protected by NAC. HFF infected with parental RH strain parasites were treated with vehicle, monensin (MO, 1 ng/mL), or myxothiazol (MYX, 50 ng/mL) in the presence or absence of NAC (50 μM). (**a**) Immunofluorescence staining with a mouse monoclonal antibody to the parasite F_1_B ATPase was used to visualize parasite mitochondrial morphology. (**b**) Thetiming of NAC treatment was assessed for the ability to protect parasite mitochondria from disruption. Parasites were treated following the timing schemes illustrated to the left of the bar graph. 50 vacuoles were quantified for each treatment scheme and the data represents 3 independent experiments. (**c**) Parasites were treated with myxothiazol with or without NAC for 24 hours to assess mitochondrial morphology after inhibition of the electron transport chain. Student’s t-test was used for statistical comparison, where **p < < 0.001 and *p < 0.033. Scale bars equal 2 μm.

Previously, we showed that prolonged monensin treatment resulted in altered mitochondrial morphology, a phenotype that is completely penetrant but entirely reversible after 24 hours of treatment^16^. Thus, alteration of mitochondrial morphology can be used as readout for monensin-driven effects that occur prior to death. To investigate the involvement of ROS in this phenomenon, intracellular parasites were vehicle or monensin treated in the presence or absence of NAC. Following 24 hours of treatment, parasites were fixed and examined by immunofluorescence microscopy for altered mitochondrial morphology as observed by staining for the F_1_B ATPase, which is located in the inner mitochondrial membrane (Bradley, unpublished). A variety of morphologies were observed following monensin treatment: *intact* morphology is indicative of healthy mitochondria with the characteristic annular appearance and little to no branching; *ribbon* morphology presents as highly disorganized strands of mitochondria that are difficult to distinguish as individual organelles; and *punctate* morphology is observed as completely disrupted mitochondria that appear to be multiple fragments of mitochondrial material (Fig. 3a). For quantification purposes, these morphologies were classified as intact or disrupted (which includes both the ribbon and punctate morphologies combined). To gain a better understanding of the extent of mitochondrial remodeling, vacuoles displaying either intact or disrupted morphology were enumerated for the various treatment conditions. Vehicle and NAC treated parasites possessed greater than 97% intact mitochondria (Fig. 3b, first and second bars). Upon monensin treatment, intact mitochondria are no longer observed such that all mitochondria display a disrupted morphology (Fig. 3b, third bar). However, co-treatment of parasites with monensin and NAC partially protects against the mitochondrial remodeling. The proportion of intact mitochondria significantly increases from 0.67% ± 1.15% with monensin treatment to 19% ± 1.41% with monensin plus NAC treatment (Fig. 3b, fourth bar). Furthermore, the number of punctate mitochondria in monensin treated parasites is dramatically reduced by 30% in the presence of NAC (reduced from 39% to 6%). The effect of monensin on the morphology is unlikely due to a generalized drug response as treatment of parasites with the cytochrome bc1 complex inhibitor myxothiazol at its IC_100_^46^,^47^ had no effect on mitochondrial morphology, yielding proportions of intact mitochondria similar to those of the vehicle and NAC controls (Fig. 3c).

It appears that scavenging ROS with the antioxidant NAC abrogated the monensin-induced disruption of mitochondrial morphology, providing evidence that the morphological disruption of the mitochondrion during monensin treatment is related to the generation of an oxidative stress. To elaborate on the protective effect of NAC and determine whether the timing of antioxidant addition was critical to the protection, we devised a treatment scheme that evaluated pre-treatment versus post-treatment. Intracellular parasites were treated with monensin alone for 12 hours, at which time drug medium was removed and replaced with growth medium containing both monensin and NAC for an additional 12 hours of treatment. In this post-treatment scheme, the addition of NAC after parasites had already been treated with monensin for 12 hours offered no protection against the drug-induced mitochondrial remodeling (Fig. 3b, fifth bar). We next assessed the protective effect of pre-treatment with NAC. Parasites were treated for 12 hours with NAC alone, washed, and incubated with monensin alone for an additional 12 hours. This pre-treatment significantly increased the percent of vacuoles displaying intact mitochondria from 1.5% in the monensin alone condition (Fig. 3b, sixth bar) to 33% in the NAC pre-treated condition (Fig. 3b, seventh bar). Once an antioxidant state was established via NAC pre-treatment, no further protection was afforded by allowing NAC to remain in the monensin treatment medium (Fig 3b, eighth bar). These data indicate that NAC must be present either before or concurrently with treatment to impart protection against the monensin-induced mitochondrial disruption.

### Monensin treatment results in a sustained oxidative stress in *T. gondii*

**Figure 4:**
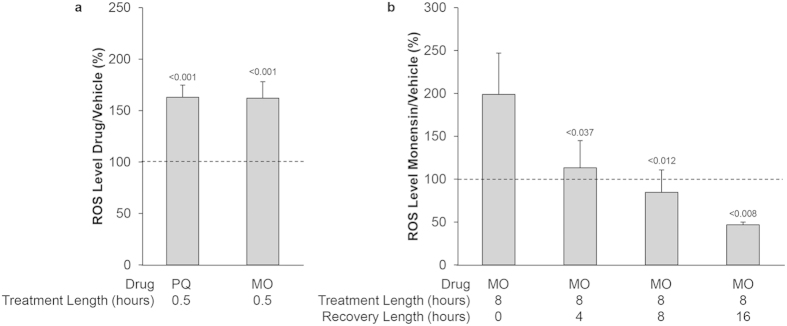
Monensin generates a sustained oxidative stress in *T. gondii*. HFF infected with the RHΔpLuc parasite strain were treated with vehicle, paraquat (PQ, 100 μM), or monensin (MO, 1 ng/mL) for 30 minutes (**a**) or 8 hours (**b**). Reactive species were measured with the OxiSelect ROS/RNS kit and were normalized relative to luciferase activity. (**b**) Parasites were treated for 8 hours followed by increasing recovery times, as indicated. Data are presented as percent vehicle treated parasites ± SD from 3 independent experiments. Statistical analysis provided by student’s t-test. Dotted line represents the vehicle treated parasites set to 100%.

While the findings above are suggestive of an oxidative stress, the use of the antioxidant NAC is not a direct assessment of the production of reactive oxygen species. Therefore, we sought to directly measure reactive species during monensin treatment of *T. gondii.* Our approach was to monitor the production of ROS with the *in vitro* ROS/RNS detection assay OxiSelect, which uses of a derivative of dichlorofluorescein that is non-fluorescent when reduced, but fluoresces in the green channel when oxidized^48^. To normalize our results across conditions, we employ a transgenic parasite line (RHΔpLuc) that expresses the luciferase enzyme^49^ whose activity can be used to control for parasite numbers. In brief, intracellular parasites were vehicle or drug treated for 30 minutes with monensin or paraquat, a quaternary ammonium bipyridyl herbicide that has been shown to generate reactive oxygen species in the related parasite *Plasmodium falciparum*^50^ and thus serves as a positive control. After treatment, parasites were manually released and filtered away from host cell debris. Levels of ROS in the lysate of equal number of parasites from each culture were detected by fluorescence and normalized with the results of luciferase measurements (which was consistent across samples correlating tightly with number of parasites used). As anticipated, paraquat caused a 62.93% ± 11.86% increase in fluorescence over vehicle treated parasites (Fig. 4a). Similarly, *T. gondii* treated with monensin also resulted in a 62.06% ± 16% (p < 0.001) increase in fluorescence compared to vehicle treated parasites (Fig. 4a). These data indicate that monensin is capable of generating reactive oxygen species very rapidly during drug treatment. However, reactive species can be unstable and short-lived; thus, we assessed whether the oxidative stress was sustained over a greater period of time. The *in vitro* ROS/RNS detection assay was again utilized to measure reactive species following 8 hours of treatment. Interestingly, the oxidative stress generated by paraquat was not observed after 8 hours, suggesting that paraquat is capable of only a brief burst of reactive species (data not shown). Conversely, 8 hours of monensin treatment produced a sustained oxidative stress that yielded a two-fold (198.84% ± 48.13%) increase in fluorescence compared to vehicle treated parasites (Fig. 4b). Not unexpectedly, the sustained oxidative stress appears to coincide with the observation that mitochondrial membrane potential is disrupted after prolonged monensin treatment, as demonstrated by reduced MitoTracker staining in parasites that display punctate mitochondrial morphology (Supplementary Fig. S2). Furthermore, parasites treated for 8 hours and subsequently allowed to recover in the absence of monensin had levels of reactive species that diminished in a time-dependent manner (Fig. 4b). These results provide direct evidence for the generation of reactive species during monensin treatment, and that the ensuing oxidative stress is maintained through extended periods of monensin drug challenge.

### Monensin-induced oxidative stress results in elevated levels of antioxidant response transcripts

**Figure 5:**
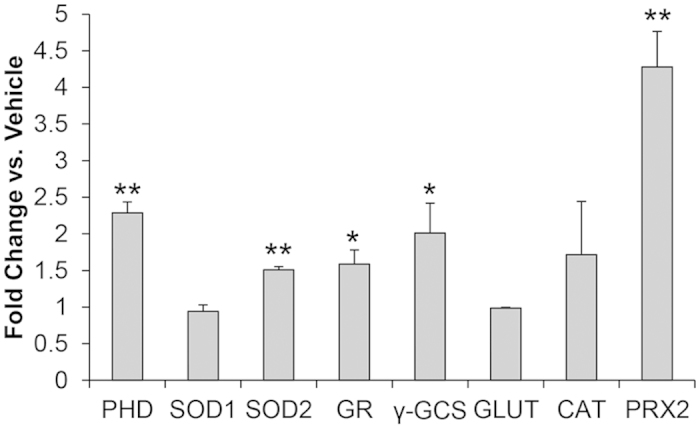
Monensin treated parasites up-regulate transcripts for antioxidant stress response genes. HFF were infected with RH parasites then vehicle or monensin (1 ng/mL) treated for 2 hours. Total RNA was isolated and used as template for cDNA synthesis. Real-time qPCR was completed using *T. gondii* specific primers for genes: PHD-finger domain-containing protein (PHD), superoxide dismutase 1 and 2 (SOD1 and SOD2), glutathione reductase (GR), γ-glutamylcysteine synthetase (γ-GCS), glutaredoxin (GLUT), catalase (CAT), and peroxiredoxin 2 (PRX2). Data were normalized using primers for the *T. gondii* 60S ribosomal protein L29 and are presented as fold change ± SD. Student’s t-test was used for statistical analysis, where **p < 0.008 and *p < 0.05. Data presented are averaged from experiments performed in triplicate.

Having established that monensin treatment creates a sustained oxidative stress, we next explored how *T. gondii* might respond to such a direct ROS assault. In general, the antioxidant response encompasses numerous proteins that function in concert to remove the harmful reactive species and/or counteract existing damage^51^. Accordingly, we hypothesized that *T. gondii* would up-regulate the expression of genes that are involved in the antioxidant stress response to contend with the continuous oxidative stress caused during monensin exposure. Quantitative RT-PCR was used to assess changes in transcript levels for a variety of genes that have been demonstrated to participate in the antioxidant response in other organisms and presumably function in *T. gondii*, including superoxide dismutase 1 (SOD1, TGGT1_316310)^52^ and 2 (SOD2, TGGT1_316330)^53^,^54^, glutathione reductase (GR, TGGT1_246920), γ-glutamylcysteine synthetase (γ-GCS, TGGT1_232590), glutaredoxin (GLUT, TGGT1_227150), catalase (CAT, TGGT1_232250)^54^,^55^, and peroxiredoxin 2 (PRX2, TGGT1_266130)^54^. As controls we used a PHD-finger domain-containing protein (PHD, TGGT1_234900), which was previously observed to be up-regulated during prolonged monensin treatment^15^, as well as ribosomal protein L29 (RPL29, TGGT1_234550), which was known not to change levels^15^. Cells infected with *T. gondii* were vehicle or monensin treated for 2 hours, and qRT-PCR was performed from the total RNA isolated from each sample. Congruent with previous data, monensin treatment resulted in elevated transcript levels of PHD (2.28 ± 0.15 fold compared to vehicle) (Fig. 5). Transcript levels for glutaredoxin (0.99 ± 0.01 fold) and SOD1 (0.94 ± 0.09) were unchanged. In contrast, SOD2, GR, γ-GCS, CAT, and PRX2 transcripts were all elevated after drug treatment 1.51 ± 0.04, 1.59 ± 0.19, 2.01 ± 0.41, 1.71 ± 0.73, and 4.28 ± 0.49 fold, respectively (Fig. 5). In conjunction, these results indicate that *T. gondii* reacts to the oxidative stress generated during monensin challenge by increasing the levels of transcripts for several genes involved in the antioxidant stress response (SOD2, GR, γ-GCS, CAT, and PRX2).

### Over-expression of antioxidant response genes abrogates the monensin-induced *T. gondii* mitochondrial remodeling

**Figure 6:**
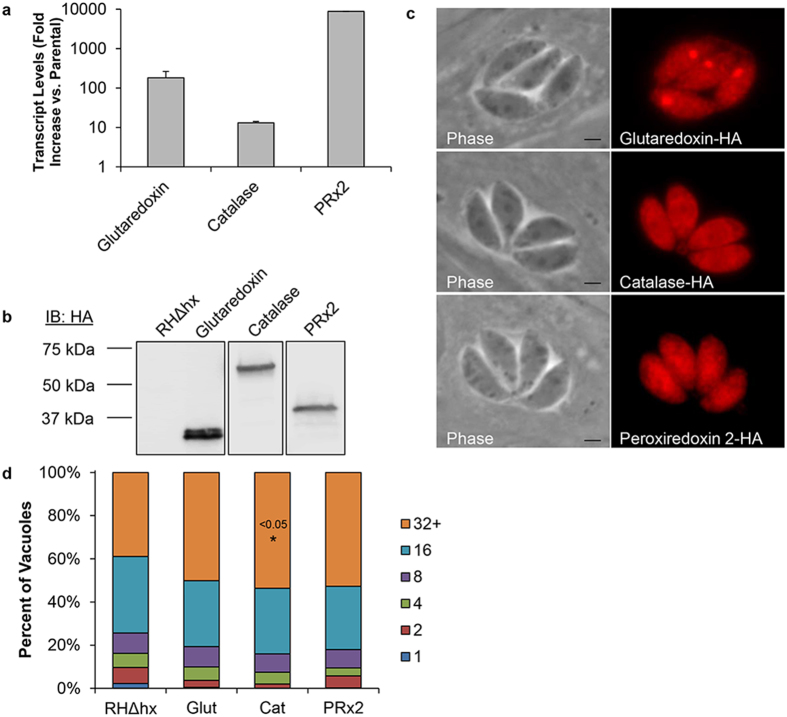
Generation of antioxidant over-expressing parasites. Transgenic parasites were created in the RHΔhxgprt background strain using plasmids designed to over-express HA-tagged versions of glutaredoxin (Glut), catalase (Cat), or peroxiredoxin 2 (PRx2) under the control of the *gra1* gene promoter. (**a**) Over-expression was verified using quantitative real-time PCR to measure transcript levels. (**b**) Western blot analysis was used to confirm protein production and that the resultant proteins were the appropriate size. (**c**) Immunofluorescence assays were performed for protein localization, utilizing a rabbit monoclonal antibody against the HA tag and an Alexa Fluor 594 secondary antibody. (**d**) General parasite replication was determined by doubling assay. Confluent HFF in 24-well plates were infected with the indicated parasite lines for 5 hours before the medium was replaced with fresh growth medium. Parasites were allowed to grow for 32 hours before being methanol-fixed and stained with DiffQuick. The number of individual parasites per vacuole was enumerated for at least 50 vacuoles. Data is presented as percent of vacuoles from 4 experiments. Student’s t-test was employed for statistical analysis. Scale bars equal 2 μm.

Our data indicate that monensin treatment of *T. gondii* results in the generation of an oxidative stress and that the parasite responds by increasing transcript levels for genes involved in the antioxidant stress response. Previous reports demonstrated that over-expression of catalase protected *T. gondii* against exogenous hydrogen peroxide challenge and oxidative injury^54^. Therefore, we reasoned that *T. gondii* could be protected against the consequences of monensin-induced oxidative stress through over-expression of antioxidant response genes. Thus, we generated multiple transgenic parasite lines that expressed ectopic copies of hemagglutinin-tagged glutaredoxin (Glut, TGGT1_227150), catalase (Cat, TGGT1_232250)^55^, or peroxiredoxin 2 (PRx2, TGGT1_266130)^54^ under the control of the *Toxoplasma gra1* promoter, which exhibits robust transcription^56^. Transcription, translation, and localization for each recombinant protein were determined via qRT-PCR, western blotting, and immunofluorescence imaging, respectively (Fig. 6a–c). Importantly, all three strains significantly overexpressed the corresponding gene in comparison to endogenous levels of the transcripts (Fig. 6a). Since there are no reagents to detect the endogenous protein, we are unable to determine the level of overexpression at the protein level. Nonetheless, significant amounts of epitope-tagged exogenous protein of the expected size are detected in each of the strains (Fig. 6b). Catalase and peroxiredoxin 2 have been described previously to localize to the parasite cytosol^54^,^55^, which we confirm here (Fig. 6c). For glutaredoxin we observed cytosolic staining with a very strong association with the apicoplast, a relic plastid of endosymbiotic origin with no photosynthetic activity (Fig. 6c). After establishing clonal populations, a doubling assay was performed to determine whether over-expression of any of the genes altered parasite replication by enumerating the number of parasites per vacuole after 32 hours of growth (Fig. 6d). Compared to the parental line, the transgenic parasite lines appear to have a slight growth advantage showing a greater proportion of vacuoles containing 32 parasites. Nonetheless, this difference is only statistically significant for the catalase over-expressing line (Fig. 6d). Thus, overexpressing any of these three genes did not result in any significant fitness defect or advantage under normal growth conditions.

**Figure 7:**
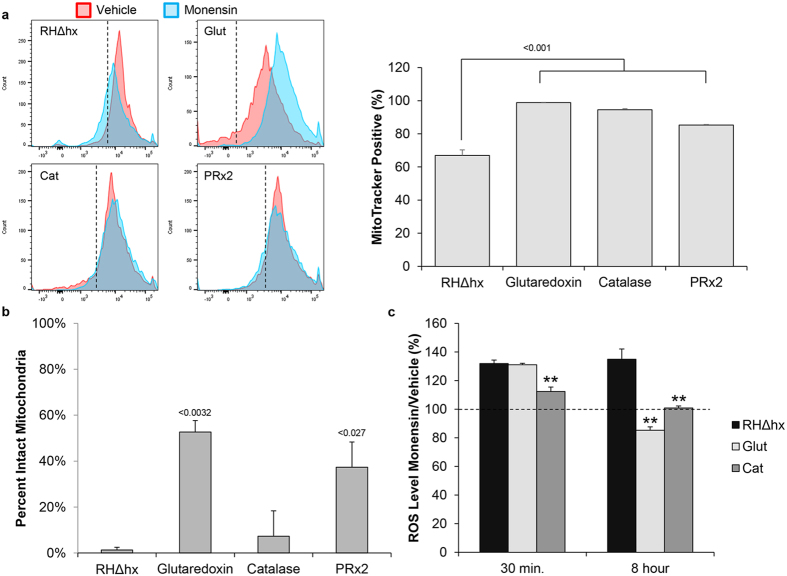
Over-expression of glutaredoxin and peroxiredoxin 2 protect the mitochondrion from monensin-induced disruption. (**a**) Flow cytometric analysis and quantification of parental and antioxidant over-expressing parasites vehicle and monensin treated for 5 hours followed by MitoTracker staining. Histograms are representative of 3 independent experiments. Quantification of MitoTracker positive parasites by flow cytometry was completed where the threshold was set at 90% of total event counts. Data presented represents 3 independent experiments. Error bars are standard deviation and statistical analysis was performed using student’s t-test where *p < 0.01. (**b**) Immunofluorescence assays were utilized to examine mitochondrial morphology in the parental RHΔhx parasites as well as the antioxidant over-expressing parasite lines glutaredoxin, catalase, and peroxiredoxin 2 (PRx2) following 24 hours of monensin (1 ng/mL) treatment. The morphology was based on the staining pattern of the F_1_B ATPase protein localized in the inner mitochondrial membrane. Only the proportions of vacuoles containing parasites with intact mitochondrial morphology are presented. Data are represented as percent ± SD and are the average of at least 50 vacuoles for each strain from 3 independent experiments. Statistical analysis provided by student’s t-test. (**c**) Reactive species were measured utilizing the OxiSelect ROS/RNS kit following either 30 minutes or 8 hours of monensin (1 ng/mL) treatment in the parental (RHΔhx) and glutaredoxin (Glut) over-expressing lines. Reactive species levels were normalized against total parasite counts. Data are presented as percent vehicle treated parasites ± SD. Results represent 4 independent replicates and statistical comparisons were made using the student’s t-test, where **p < 0.0001. Dotted line represents the vehicle treated parasites set to 100%.

As we observed that NAC could mitigate the monensin-induced decreases in mitochondrial membrane potential, we next wanted to assess whether the over-expression of antioxidant proteins could also protect Δψm. Flow cytometry was utilized to monitor changes is MitoTracker fluorescence following 5 hours of monensin treatment (Fig. 7a). As expected, drug challenged parental parasites (RHΔhx) displayed reduced MitoTracker staining as compared to vehicle treatment, 66% vs. 90%, respectively (Fig. 7a). In contrast, over-expression of the antioxidant proteins prevented the loss in mitochondrial membrane potential observed in the parental parasite line (Fig. 7a). Over-expression of glutaredoxin and catalase increased the proportion of MitoTracker positive parasites following monensin treatment by 9% and 5% over vehicle treatment, respectively (Fig. 7a). Though statistically significant, the 5% reduction in MitoTracker positive parasites from vehicle treatment in the peroxiredoxin 2 line (Fig. 7a) is likely of little consequence (see below). Altogether, over-expressing the antioxidant proteins is capable of protecting against the monensin-induced reduction in mitochondrial membrane potential.

Finally, each parasite line was assessed for its sensitivity to monensin using mitochondrial remodeling as a readout. All vehicle treated parasites maintained intact mitochondrial morphology as expected (data not shown). Expectedly, only 1.33% ± 1.15% of parental parasites exhibited intact mitochondrial morphology (Fig. 7b). While the proportion of intact mitochondria increased slightly to 7.33% ± 11.02% for the catalase over-expressing line, it was not statistically significant (p > 0.2). In contrast, over-expression of glutaredoxin or peroxiredoxin 2 significantly protected against monensin-induced mitochondrial remodeling, yielding 52.67% ± 5.03% and 37.33% ± 11.02% intact mitochondria, respectively (Fig. 7b). Furthermore, over-expression of glutaredoxin and peroxiredoxin 2 decreased the percentage of parasites showing punctate mitochondria from 77.33% ± 6.11% for the parental strain to 10.00% ± 0.00% (p < 0.003) for the glutaredoxin over-expressor and 17.33% ± 18.15% (p < 0.05) for the peroxiredoxin 2 over-expressing line. Together, these results demonstrate that over-expressing antioxidant response genes, such as glutaredoxin and peroxiredoxin 2, provides a protective effect against monensin-induced mitochondrial remodeling.

### Glutaredoxin and catalase over-expression reduces monensin-induced reactive species

At present, our data indicates that an oxidative stress is involved, in part, in the mode of action for monensin, and that reducing this oxidative stress offers protection from the drug’s deleterious effects. Since we observed that the greatest amount of protection was provided by over-expression of glutaredoxin, while only slight protection was offered by catalase over-expression, we decided to investigate whether elevated levels of these antioxidant response proteins were indeed functioning to reduce the oxidative stress caused by monensin treatment, thereby strengthening the connection between monensin-induced reactive species and altered mitochondrial biology. We again utilized the OxiSelect kit to measure the levels of reactive species following either 30 minutes or 8 hours of monensin treatment of the parental as well as glutaredoxin and catalase over-expressing parasite lines. At 30 minutes of drug treatment, the levels of reactive species were equally elevated in the parental and glutaredoxin over-expressing lines, displaying 131.9% ± 2.41% and 130.98% ± 1.09% of the levels of vehicle treated parasites, respectively (Fig. 7c). While the levels of reactive species were also elevated in the catalase over-expressing parasites (112.4% ± 3.08%) compared to vehicle treatment, these levels were lower than in the parental and glutaredoxin parasites (Fig. 7c). However, after 8 hours of monensin treatment, we observed that the over-expression of glutaredoxin dramatically reduced the oxidative stress in these parasites. The parental line exhibited reactive species levels upon monensin treatment of 134.9% ± 7.12% in relation to untreated controls; whereas, the levels of reactive species produced by monensin in the glutaredoxin over-expressing parasite line were 85.24% ± 2.39% of that in the untreated control (Fig. 7c). Likewise, the catalase over-expressing parasites demonstrated reduced ROS levels after 8 hours of monensin treatment (100.8% ± 1.4%) compared to parental parasites, albeit to a lesser extent than the glutaredoxin parasites (Fig. 7c). These data indicate that glutaredoxin and catalase can function to alleviate oxidative stress, but their ability to protect *T. gondii* from the effects of the reactive species produced during sustained monensin treatment appears to correlate to their overall capacity to reduce ROS levels.

### Disruption of the Golgi apparatus accompanies mitochondrial fragmentation following monensin treatment

**Figure 8:**
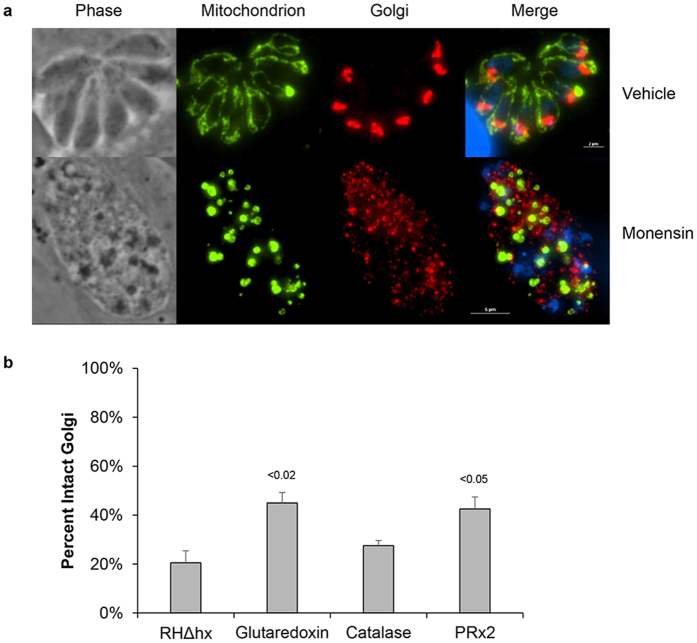
Monensin treatment fragments the Golgi apparatus but is diminished by over-expression of glutaredoxin and peroxiredoxin 2. (**a**) Immunofluorescence microscopy of HFFs infected with parental RH strain parasites treated with vehicle or monensin (1 ng/mL) for 24 hours. The mitochondrion and Golgi were visualized using antibodies to the F_1_B ATPase and sortilin-like receptor proteins, respectively. (**b**) The extent of Golgi disruption was quantified by counting 50 vacuoles for each parasite strain and treatment condition. Data are presented as percent intact Golgi ± SD and represent 3 experiments.

While the mitochondrion appears to by the predominant target of the drug monensin, it should be noted that observations gleaned from electron microscopy of mammalian fibroblast cells indicates that the Golgi apparatus is also subject to the drug’s effects^13^,^14^. Though we previously examined the organization of various organelles in monensin-treated parasites^16^, including the nucleus, apicoplast, and plant-like vacuole, and demonstrated that these were unperturbed, it remained to be determined whether the *T. gondii* Golgi was affected by monensin. The Golgi apparatus of *T. gondii* is situated above the nucleus oriented toward the apical side within tachyzoite parasites^57^. Under normal growth conditions, the nucleus and Golgi stacks are often observed to be encircled by the parasite mitochondrion (Fig. 8a) as demonstrated via immunofluorescence microscopy employing an antibody to the sortilin-like receptor^58^. However, upon monensin treatment, the architecture of the Golgi is completely lost, and staining can be observed as punctate vesicles diffusely distributed throughout the parasite cytoplasm (Fig. 8a). Interestingly, just as the mitochondrion can recover after drug withdrawal, so too can the Golgi apparatus (data not shown). As we demonstrated that the mitochondrion is protected from disruption in the parasite lines ectopically expressing the antioxidant genes, we next sought to determine whether their over-expression could similarly protect the Golgi from monensin-induced disruption. Congruent with the results for the mitochondrion, the glutaredoxin (45.00% ± 4.24%) and peroxiredoxin 2 (42.50% ± 4.95%) expressing parasites maintained a greater proportion of intact Golgi in the presence of monensin as compared to the parental parasites (20.50% ± 4.95%) and catalase (27.50% ± 2.12%) expressing parasite line (Fig. 8b). These data indicate that the oxidative stress induced by monensin treatment is a large contributing factor to the overall deleterious effects of the drug.

### Protecting the mitochondrion and Golgi translates to better parasite growth in the presence of monensin

**Figure 9:**
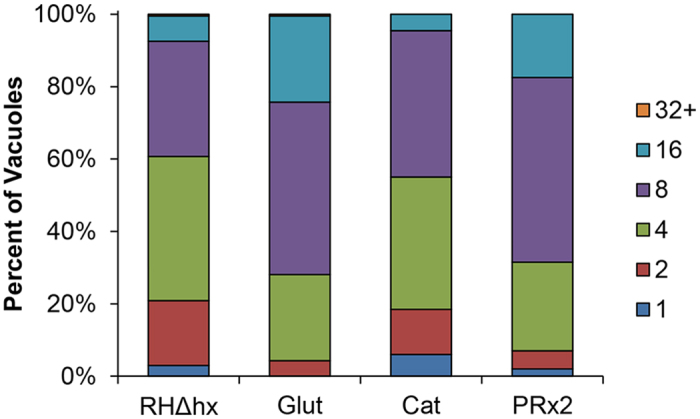
Over-expression of glutaredoxin and peroxiredoxin 2 allows parasites to resist monensin-induced growth arrest. Doubling assays were employed to assess the ability of the transgenic parasite lines to grow in the presence of monensin (0.75 ng/mL). Parental RHΔhx parasites and the glutaredoxin (Glut), catalase (Cat), and peroxiredoxin 2 (PRx2) over-expressing lines were infected into HFF and grown for 32 h before fixing with methanol and staining with DiffQuick. The number of individual tachyzoites per vacuole was enumerated for at least 50 vacuoles from 4 separate experiments. The data are presented as percent ± SD.

Thus far, our data demonstrate that over-expression of certain antioxidant genes can protect against the monensin-induced mitochondrial and Golgi disruption (Figs 7b and 8), and in the case of glutaredoxin, this may be a direct result of its antioxidant function and ability to significantly reduce reactive oxygen species (Fig. 7c). However, it’s unclear whether protecting the mitochondrion and Golgi apparatus would have a direct positive outcome for the parasites during exposure to monensin, which ultimately results in parasite death. Hence, we sought to determine how over-expression of the antioxidant genes would affect the ability of parasites to replicate in the presence of monensin compared to the parental parasite line. We performed a doubling assay, growing parasites in the presence of 0.75 ng/mL monensin for 32 hours, at which point the number of parasites per vacuole was enumerated. In all parasite lines tested, it was observed that monensin impacted the growth rate as compared to vehicle treated parasites. After 32 hours of intracellular growth in vehicle medium, parental and transgenic lines had more than 75% of vacuoles with 16 or more parasites (Fig. 6d); whereas, in monensin treated parasites of all strains, the majority of vacuoles contained 8 or fewer parasites at the same time-point (Fig. 9). Parasite lines over-expressing either glutaredoxin or peroxiredoxin 2 had significantly better growth in the presence of monensin as compared to that of the parental or catalase over-expressing parasite lines. In the parental and catalase over-expressing parasite lines, 39.5% and 45% of vacuoles contained more than 8 parasites, respectively; whereas, 68.5% and 75% of vacuoles in the peroxiredoxin 2 and glutaredoxin transgenic parasite lines contained greater than 8 parasites, respectively (Fig. 9). The slight, but not statistically significant, growth advantage seen for the antioxidant lines in normal growth conditions (Fig. 6d) cannot account for the statistically significant increase in growth rate in the presence of monensin for the peroxiredoxin 2 and glutaredoxin over-expressing parasites. In vehicle control, we see only a 1.3 and 1.4 fold increase in the number of vacuoles with more than 32 parasites for the glutaredoxin and peroxiredoxin 2 over-expressing parasites, respectively, as compared to the parental strain. By contrast, the fold increase in the number of vacuoles with more than 16 parasites in the presence of monensin is 3.6 and 2.5 for the glutaredoxin and peroxiredoxin 2 over-expressing parasites, respectively. Importantly, the catalase over-expressing strain, which was the only one with a significant growth advantage in vehicle conditions, did not exhibit any difference in growth with monensin relative to the parental strain. It is important to note that the genes that protect against the monensin-induced growth arrest, glutaredoxin and peroxiredoxin 2, are the same that protect the mitochondrion and Golgi apparatus from morphological disruption and significant ROS production, indicating a connection between these two phenomena.

## Discussion

**Figure 10:**
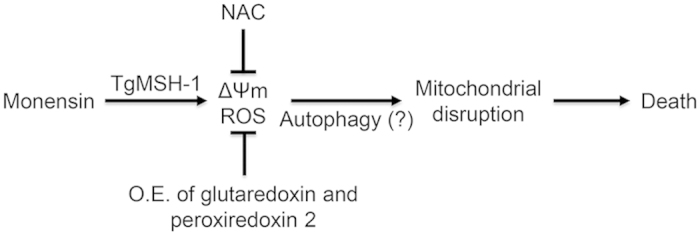
Model for the mechanism of action for the anti-parasitic drug monensin. Monensin treatment results in decreased mitochondrial membrane potential (ΔΨm) and disruption of the mitochondrial morphology, which ultimately leads to parasite death. The deleterious effects of monensin can be abrogated by the addition of the antioxidant compound N-acetyl-cysteine (NAC) or by the over-expression (O.E.) of glutaredoxin or peroxiredoxin 2, suggesting the involvement of an oxidative stress. TgMSH-1 appears to be the upstream mediator of the effects of monensin on the mitochondrion as well as being involved in signaling the induction of autophagy.

Here we provide evidence that monensin targets the parasite mitochondrion, disrupting mitochondrial membrane potential, and dramatically altering the morphology of this important organelle. Moreover, the Golgi apparatus is affected by monensin treatment and is subject to fragmentation. The observed effects on the Golgi apparatus may have consequences for proper inner membrane complex (IMC) formation^59^ and overall parasite morphology, as reflected by parasite swelling^8^,^18^. Furthermore, we demonstrate that monensin treatment elicits an oxidative stress that is abrogated by either the addition of an antioxidant compound or over-expression of antioxidant stress response genes. Interestingly, when parasites are able to mitigate the monensin-induced oxidative stress through the over-expression of antioxidant genes, they display higher proportions of vacuoles with intact mitochondrial and Golgi morphology and are able to progress through the growth arrest normally elicited by monensin treatment. We present a model (Fig. 10) where monensin treatment generates reactive oxygen species as well as induces the loss in membrane potential and eventual disruption of the mitochondrial architecture. These events appear to be mediated through the DNA repair protein TgMSH-1, as studies with a parasite line mutated for this protein are protected from mitochondrial remodeling^16^ and do not demonstrate the loss in membrane potential (unpublished data). The induction of autophagy is signaled by the TgMSH-1-dependent cell cycle arrest and may be concomitantly regulated by the monensin-generated ROS through post-translational oxidation of autophagy proteins as well as their upstream regulators^60^,^61^,^62^,^63^. While parasites are able to mount an antioxidant stress response, it is not sufficient to mitigate the effects of prolonged monensin treatment and parasites ultimately die.

The observed effects of monensin treatment on *Toxoplasma gondii* are strikingly similar to those demonstrated in higher eukaryotic systems. Following monensin challenge, we demonstrated that an oxidative stress is induced, mitochondrial membrane potential is reduced, and the mitochondrion is morphologically disrupted. Based on our observations and those of other groups, a possible explanation for the effects on the mitochondrion is that monensin shuttles protons across the mitochondrial membranes, disrupting the electrochemical gradient required for proper organelle function. Such an ionic imbalance results in the downstream consequences demonstrated here and elsewhere. Additional support for our overall model is provided by studies in mammalian cells utilizing CCCP and NAC. It was demonstrated that CCCP treatment leads to generation of reactive oxygen species, dissipation of mitochondrial membrane potential, and fragmentation of the mitochondria, all of which can be blocked by the addition of NAC^64^,^65^,^66^. Similar cellular consequences have been described to occur after monensin treatment in a variety of mammalian cells^13^,^14^,^27^,^29^,^31^,^32^. However, it is important to note that the drug-induced effects elicited in *T. gondii* resulted after monensin challenge using concentrations 50 times less than those required to affect mammalian cells. One possible explanation for the disparity in drug concentration necessary to alter mitochondrial function may lie in the disproportion of mitochondrial abundance. Mammalian cells contain hundreds to thousands of mitochondria, which are maintained through a dynamic process of remodeling to repair or selectively remove damaged mitochondria^67^,^68^,^69^. By contrast, *T. gondii* possesses a single mitochondrion, suggesting the parasite is bereft of some of the protective processes available to higher eukaryotic cells. Such a fundamental difference in this critical organelle is believed to be a significant liability and represents an excellent target for therapeutic development^70^.

Post-monensin treatment, we first observe ROS production at 30 minutes, decreased ΔΨm at 5 hours, and peak mitochondrial disruption at 24 hours, though the earliest observable remodeling occurs after 8 hours of monensin challenge. While the timing of our experiments and observations suggests that an initial oxidative insult occurs prior to the membrane potential disruption and mitochondrial remodeling, we cannot exclude the possibility that an unappreciated decrease in mitochondrial membrane potential occurs immediately upon monensin treatment that results in the generation of the measured ROS. Indeed, the oxidative stress we observed may be attributable to the ionophoric activity of monensin. By disrupting the ionic homeostasis in the mitochondrion, monensin treatment is likely uncoupling oxidative phosphorylation and dissipating the membrane potential, which ultimately leads to the generation of reactive oxygen species. The phenomenon of ionophore-induced ROS production has been observed with beauvericin, a compound produced by toxigenic fungi^71^,^72^,^73^. Moreover, it has been reported that an initial burst of ROS can result in a larger wave of ROS production in what could be a feed-forward loop^42^. This ROS-induced ROS production might explain the fact that the oxidative stress caused by monensin in *Toxoplasma* is not simply an immediate burst of reactive species, but a prolonged generation of ROS, as demonstrated in Fig. 4b. While an initial burst of ROS generation is also observed with paraquat, no ROS were detected at 8 hours of paraquat treatment. Interestingly, no ill effects were observed for the parasite mitochondrion following paraquat challenge and parasites are able to divide in the presence of paraquat. The contrast between the effects of monensin and paraquat suggest that it is the sustained oxidative insult that causes mitochondrial dysfunction and growth arrest. Sustained oxidative stress would be very destructive, resulting in damaged proteins, lipids, and DNA, likely contributing to the potency and lethality of monensin.

Underscoring the role of oxidative stress in the ill-effects caused by monensin is the protection rendered by overexpression of the antioxidant factors glutaredoxin or peroxiredoxin 2. An interesting point to note is that over-expression of catalase was unable to afford protection against mitochondrial disruption and slowed growth following monensin challenge. This was unexpected, as we noted no loss in mitochondrial membrane potential and lower ROS levels compared to parental parasites as well as previous observations made in parasites over-expressing catalase that showed it was protective against exogenous hydrogen peroxide treatment^54^. It is possible that the levels of expression are not enough to provide protection beyond what is rendered by the endogenous protein. Alternatively, the particular function of catalase as compared to that of either glutaredoxin or peroxiredoxin 2 might provide an explanation for our observation. Catalase is an enzyme that converts hydrogen peroxide into molecules of water and oxygen, while glutaredoxin and peroxiredoxin 2 are proteins capable of directly reducing proteins that are oxidatively damaged. Thus, it could be surmised that either monensin treatment does not result in appreciable amounts of cellular hydrogen peroxide to be eliminated by catalase, or the capacity for glutaredoxin or peroxiredoxin 2 to reverse the oxidative damage on proteins plays a more significant role in protecting these parasites. The latter is consistent with our observation that it is the persistent elevated levels of ROS that are responsible for the effects of monensin.

Although efforts to elucidate and understand the nature of the mitochondrial genome of *Toxoplasma* have yet to be fulfilled, its location within this organelle renders it highly susceptible to oxidative damage. Indeed, our initial work concerning monensin demonstrated that the drug’s effects, including cell cycle arrest and cell death, were mediated through a MutS homolog (TgMSH-1) that localizes within the parasite mitochondrion^18^. Numerous MSH proteins have been implicated in DNA mismatch repair (MMR), and their respective roles have been tightly linked with sensing DNA damage and signaling to cell cycle arrest and cell death^74^. Interestingly, in yeast, the repair of oxidatively damaged mitochondrial DNA was demonstrated to be dependent upon the MSH1 pathway^75^,^76^. Collectively, our observations suggest that monensin induces an oxidative stress that likely damages the mitochondrial genome, which is detected by TgMSH-1 and signals for the parasites to arrest and ultimately die if the damage cannot be repaired. Moreover, in the absence of TgMSH-1, any damage to the mitochondrial genome goes unnoticed, precluding cell cycle arrest and cell death, resulting in the drug-resistant phenotype observed with TgMSH-1 mutant parasite lines. If attempts to repair the damaged mitochondrial DNA are unsuccessful, one means of mitigating the deleterious effects is to selectively remove the organelle containing the damaged DNA in a process known as mitophagy^77^,^78^. As *Toxoplasma* possesses only one mitochondrion, selective removal of this entire organelle through mitophagy seems like an improbable solution to managing damaged DNA. However, we cannot exclude the possibility that fission plays a role in segregating damaged DNA into fragments destined for removal^68^,^79^. The monensin-induced mitochondrial fragmentation we observe is consistent with such a process and could in fact involve organelle remodeling and eventual destruction of the fragments that contain defective mitochondrial genomes and/or damaged proteins and lipids. To address these possibilities, we are actively investigating proteins involved in the processes of MMR and mitochondrial fission.

An inevitable consequence of aerobic metabolism is the continuous exposure to endogenously generated reactive oxygen and nitrogen species, which can be deleterious to lipids, proteins, and nucleic acids. To combat these toxic effects, the antioxidant system consists of a network of proteins that function directly to reduce oxidative damage and includes enzymes involved in the recycling of redox proteins, enzymes that participate in the synthesis of antioxidants, and enzymes that catalyze the conversion of reactive species into less harmful molecules^51^. This is of particular importance in intracellular parasites that must contend not only with the oxidative stresses produced during division and metabolism but also those generated by the host^80^. In the case of *Plasmodium falciparum*, disruption of the 2-Cys peroxiredoxin PfTPx-1 resulted in parasites that were more readily killed by paraquat and sodium nitroprusside^81^. *T. gondii* deficient for catalase exhibited increased susceptibility to exogenous hydrogen peroxide and were less virulent in mice^54^. These findings underscore the important nature of redox biology for parasite survival, and hint toward a critical system with which interference could be a viable means to target infection. Indeed, a number of studies have shown that eliciting an oxidative stress during parasitic infections can be an efficient means of killing. In fact, the anti-parasitic activity for a variety of compounds has been demonstrated to involve the generation of reactive species and mitochondrial dysfunction. In *T. gondii*, the antitumor quinolone NSC3852 generated an oxidative stress to potently inhibit growth, which was reduced in the presence of antioxidants^82^. Aryl aryl methyl thio arenes (AAMTAs) demonstrated potent anti-malarial activity against a multidrug resistant *Plasmodium yoelii* in a mouse model of infection, which was a result of oxidative damage and depletion of parasite stores of glutathione^83^. Glabridin treatment of *P. falciparum* generated reactive oxygen and nitrogen species to induce an apoptotic death^84^. Altogether, these observations and our findings that monensin’s lethal effect is in part due to ROS production, strengthen the argument for further exploring compounds that induce an oxidative stress and/or interfere with parasite redox biology as an avenue for therapeutic development against parasitic infections including *Toxoplasma gondii*.

## Materials and Methods

### Host cell and parasite maintenance and reagents

Human foreskin fibroblasts (HFF, purchased from ATCC) were grown in normal culture medium, which was Dulbecco’s modified Eagle medium (DMEM) supplemented with 10% fetal bovine serum (FBS), 2 mM L-glutamine, and 100 units penicillin/100 μg streptomycin per mL, in a humidified incubator at 37 °C and 5% CO_2_. All parasite strains were maintained by passage through HFFs in normal culture medium. For drug treatment experiments, media was supplemented with 1% FBS instead of 10%, unless otherwise stated. All drugs were acquired from Sigma. Stocks of monensin, N-acetyl-cysteine, and myxothiazol were prepared in ethanol; atovaquone and CCCP were prepared in DMSO; and paraquat was prepared in water and used at the final concentrations indicated.

### Microscopy and immunofluorescence assays

All phase and fluorescence microscopy was performed on a Nikon Eclipse 80i microscope, and images were captured and processed using NIS-Elements AR 3.0 software. All cultures were fixed with 3.5% formaldehyde. The parasite mitochondrion was detected using the mouse monoclonal antibody 5F4 (α-F_1_B ATPase) kindly provided by P. Bradley (unpublished). The parasite Golgi was detected by a rat polyclonal antibody^58^. HA-tagged proteins were visualized using a rabbit monoclonal antibody (Cell Signaling). Primary antibodies were visualized using either Alexa Fluor 594 or Alexa Fluor 488 secondary antibodies (Life Technologies). Coverslips were washed and mounted on glass slides with 3 μL Vectashield with DAPI (Vector Labs).

### MitoTracker staining for mitochondrial membrane potential

HFFs seeded on glass coverslips were infected with RH strain parasites expressing the green fluorescent protein (GFP). One day after infection, normal culture medium was replaced with culture medium containing monensin (1 ng/mL) or atovaquone (10 nM) with or without N-acetyl-cysteine (NAC, 50 μM) for 5 or 24 hours. Following drug treatment, cells were washed and incubated with medium containing 200 nM MitoTracker Red CMXRos (Life Technologies) for 25 minutes in a 37 °C incubator. After staining, cultures were washed in PBS and fixed with 3.5% formaldehyde in PBS for 20 minutes. For visualizing the mitochondrial membrane potential in extracellular parasites, infected HFFs in T-25 flasks were drug treated then scraped and syringe lysed with a 30G needle and host cell debris was removed by filtration through 3.0 μm pore-size membrane filters (Whatman). MitoTracker staining was completed as before but with parasites in suspension. Parasites were pelleted and washed 3x with PBS before fixation. After fixation, 10 μL of the parasite suspension was air-dried onto glass coverslips then mounted on glass slides and visualized as described above. Parasites displaying bright, reduced, and no red fluorescent MitoTracker staining were enumerated. At least 100 parasites were counted for each drug treatment and results represent 3 independent experiments.

MitoTracker, JC-1, and TRME staining were used in conjunction with flow cytometric analysis to assess mitochondrial membrane potential. Intracellular parasites were treated for 5 hours in 1% FBS medium containing vehicle, monensin (0.75 ng/mL), or CCCP (1 μM) with or without NAC (50 μM). Following treatment, parasites were manually released from host cells by syringe lysis and filtration to remove cellular debris. MitoTracker, JC- 1, or TMRE was added to the drug-containing medium at a final concentration of 50 nM and incubated for 45 minutes at 37 °C. MitoTracker stained parasites were washed with pre-warmed PBS, pelleted, and fixed in 70% ethanol/PBS overnight at −20 °C. Prior to analysis, parasites were pelleted and resuspended in 50 mM Tris (pH 7.5), RNase cocktail (Ambion), and 2 μM Sytox green (Life Technologies). JC-1 and TMRE stained parasites were left in the drug-containing medium minus phenol red. Parasites were analyzed using a BD LSRII (561 nm laser) flow cytometer (BD Biosciences) and FlowJo V10 software (Tree Star Inc., Ashland, OR). The threshold for MitoTracker and TMRE positive parasites was set at 90% of event counts^85^. JC-1 aggregate formation was assessed as an indicator of membrane potential.

### Immunofluorescence assays for mitochondrial and Golgi morphology

HFFs infected with parental RH strain parasites or parasites over-expressing antioxidant genes were vehicle or drug treated with either monensin (1 ng/mL) or myxothiazol (50 ng/mL) in the presence or absence of NAC (50 μM) for 24 hours. Following drug treatment, cells were processed for imaging as described above. The proportions of vacuoles possessing parasites displaying intact or disrupted mitochondrial and Golgi morphologies were quantified. At least 50 vacuoles were enumerated for each parasite strain and treatment condition, and the results represent 3 independent experiments.

### Measuring reactive oxygen species and luciferase activity

HFFs were infected with a parasite strain that expresses the light-emitting firefly luciferase (Luc) protein, RHΔpLuc^49^, which allows for the control of parasite number. Twenty-four hours after infection, the medium was replaced with serum-free medium containing either paraquat (100 μM) or monensin (1 ng/mL). Following 30 minutes or 8 hours of drug treatment, host cells were scraped and manually disrupted by passing through a 27G needle and syringe to release intact parasites. Parasites were collected by centrifugation and equal numbers of parasites were resuspended in parasite lysis buffer (150 mM NaCl, 50 mM Tris-HCl, 0.1% NP-40). Reactive oxygen species (ROS) were measured using the OxiSelect *In Vitro* ROS/RNS Assay Kit (Cell Biolabs, Inc.) per manufacturer’s instructions. Briefly, 50 μL of parasite lysate was added to the wells of a black-walled 96-well plate (Greiner Bio-One, Sigma) followed by 50 μL of Catalyst reagent and then incubated at room temperature for 5 minutes. 100 μL of the DCFH solution was added to all wells, covered with foil, and incubated in the dark at room temperature for 45 minutes. Fluorescence was measured at 480 nm/530 nm Ex/Em in a Synergy H1 microplate reader (BioTek). All samples were assayed in duplicate. Luciferase activity was measured following the ROS measurements to control for parasite number. In brief, 100 μL of Bright-Glo reagent (Promega) is added to 100 μL of the parasite lysate solution, mixed well, and luminescence is measured in the microplate reader. Samples are again assayed in duplicate, and the final results represent 3 independent experiments. Data was expressed as percent of vehicle treated parasites following normalization based on the relative luciferase activity for each parasite sample. For recovery, parasites were treated for 8 hours, followed by 0, 4, 8, or 16 hours of recovery in normal growth medium in the absence of monensin. ROS and luciferase were measured as described above.

Similarly, ROS levels were measured in parental RH parasites or the RH parasites over-expressing glutaredoxin after 30 minutes and 8 hours of vehicle or monensin treatment. Since these parasite lines do not express the luciferase gene, ROS levels were normalized to total parasite number used in the assay, which was determined by counting using a hemocytometer.

### Quantitative RT-PCR

HFFs infected with RH parasites were vehicle or drug treated with monensin (1 ng/mL) for 2 hour. The parasites were syringe lysed, and total RNA was harvested utilizing the RNeasy Plus Minikit (Qiagen). cDNA was made using the QuantiTect Reverse Transcription Kit (Qiagen). Real-time quantitative PCR was completed using *T. gondii*-specific primer pairs and Fast SYBR Green Master Mix (Applied Biosystems) in a StepOne PCR machine (Applied Biosystems). Relative quantification was determined by the comparative threshold cycle (ΔΔ*C*_*T*_) method. The cDNA quantity was normalized between samples employing primers for the *T. gondii* 60S ribosomal protein L29 (GeneID TGGT1_234550). Results presented are averages of three experiments each performed in triplicate. Likewise, transcript levels were determined for the RH parasites over-expressing the anti-oxidant genes glutaredoxin, catalase, and peroxiredoxin 2 to verify actual over-expression compared to parental RH parasites. Primers used in all qRT-PCR experiments are listed in Supplemental Table 1.

### Generation of antioxidant over-expressing parasites

Transgenic parasite lines were created to over-express the antioxidant genes for glutaredoxin (TGGT1_227150), catalase (TGGT1_232250), and peroxiredoxin 2 (PRx2, TGGT1_266130). PCR was used to generate hemagglutinin (HA)-tagged cDNA flanked by *Avr*II and *Bgl*II restriction enzyme sites. Primers used to amplify and tag the cDNAs are found in Supplemental Table 1. Restriction enzyme digestion and ligation cloning was used to insert each fragment into the pgra-HX plasmid, which carries hypoxanthine-xanthine-guanine phosphoribosyltransferase (*HXGPRT*) under the control of the *gra1* promoter as a selectable marker. Plasmids were verified through PCR, restriction enzyme, and sequencing analyses. 30 μg of *Not*I-linearized plasmid were electroporated into the parental RHΔhxgprt parasite line that lacks a functional HXGPRT gene^86^. Selection for parasites that successfully integrated the plasmid was performed in culture medium containing 50 μg mycophenolic acid and 50 μg xanthine per mL. After three rounds of selection, individual parasite clones were established from the population by limiting dilution and HA-tag positive clones were subsequently selected for outgrowth.

### Western blot analysis

Infected HFFs were passed through a 27G needle and syringe to release intact parasites, then host cell debris was filtered away and parasites were collected by centrifugation. Parasites were resuspended in RIPA buffer (50 mM Tris-HCl pH 7.4,150 mM NaCl, 0.5% sodium deoxycholate, 1% NP-40 substitute, 0.1% SDS) to prepare protein lysates. SDS sample buffer was added to protein lysates, which were then subjected to SDS-PAGE. Proteins were transferred to nitrocellulose membranes using a Semi-Dry Electroblotter (BioRad). The antioxidant proteins were detected using a rabbit α-HA primary antibody (Cell Signaling). Primary antibodies were detected by an α-rabbit IgG secondary antibody conjugated to horseradish peroxidase (Cell Signaling). Membranes were incubated with SuperSignal West Femto Chemiluminescent substrate (Pierce) for 5 minutes then imaged using a FluorChem E system machine (Protein Simple).

### Doubling Assay

Confluent HFFs in 24-well plates were infected with 1000 parasites per well for the indicated strains. The parasites were allowed to invade for 5 hours before the medium was removed and unattached parasites were washed away. Fresh medium with or without monensin (0.75 ng/mL) was added and the infection progressed for 32 hours before the cultures were fixed with methanol and stained with DiffQuick (Siemens). The number of individual tachyzoites per vacuole was enumerated in random fields of view until 50 vacuoles had been counted for each parasite strain. The data presented are the average of 4 replicates.

## Additional Information

**How to cite this article**: Charvat, R. A. and Arrizabalaga, G. Oxidative stress generated during monensin treatment contributes to altered *Toxoplasma gondii* mitochondrial function. *Sci. Rep.* **6**, 22997; doi: 10.1038/srep22997 (2016).

## Supplementary Material

Supplementary Information
