# Supplementary material for: Oxidative stress generated during monensin treatment contributes to altered Toxoplasma gondii mitochondrial function
Source: Sci Rep. 2016 Mar 15;6:22997. doi: 10.1038/srep22997 (PMC4792157; doi:10.1038/srep22997)
Supplement: Supplementary Information [file srep22997-s1.pdf]

Supplementary Information

Oxidative stress generated during monensin treatment contributes to altered *Toxoplasma gondii* mitochondrial function

Robert A. Charvat[‡] and Gustavo Arrizabalaga[‡§*]

From the [‡]Departments of Pharmacology and Toxicology and [§]Microbiology and Immunology, Indiana University School of Medicine, Indianapolis, Indiana 46202

[*]To whom correspondence should be addressed: Dept. of Pharmacology and Toxicology, Indiana University School of Medicine, 635 Barnhill Drive, Indianapolis, IN 46202, Tel: (317) 278-6355; E-mail: garrizab@iu.edu

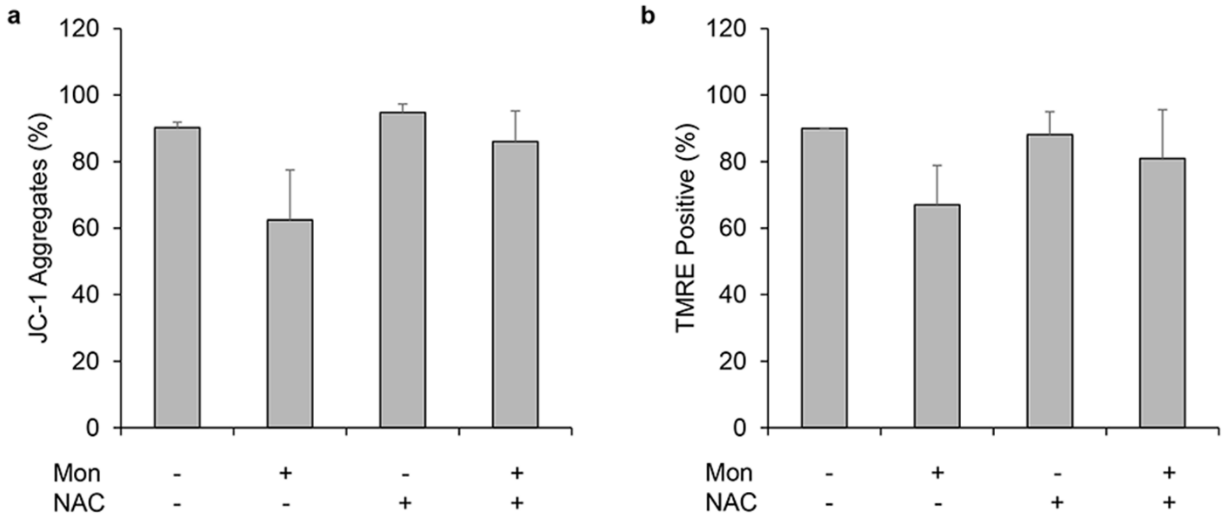

Supplementary Figure S1. **NAC protects against membrane potential dissipation caused by monensin treatment in JC-1 and TMRE stained parasites.** HFFs infected with parental RH strain parasites were vehicle or monensin (1ng/mL) treated in the presence or absence of NAC (50 µM) for 5 hours. Following treatment, parasites were manually released by syringe lysis and filtered to remove host cell debris. Parasites were washed and stained in 1% FBS DMEM (without phenol red) containing the specified drug treatments with 50 nM of either (**a**) JC-1 or (**b**) TMRE. Data presented are averaged from 2 experiments.

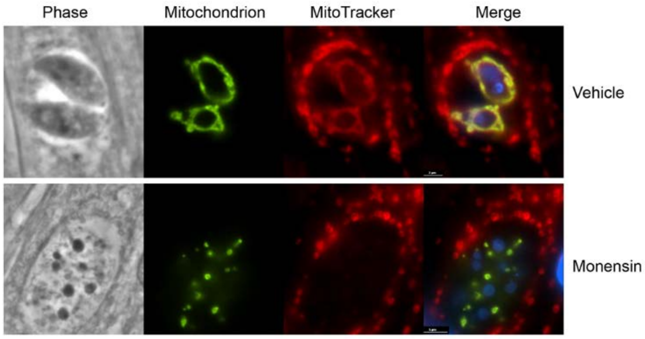

Supplementary Figure S2. **Prolonged monensin treatment results in fragmented mitochondria that do not stain with MitoTracker.** HFFs infected with parental RH strain parasites were vehicle or monensin (1ng/mL) treated for 12 hours. Cells were stained with MitoTracker (50 nM) in phenol red free medium at 37ºC for 45 minutes. Cells were washed, fixed, and stained for the mitochondrion as performed previously for standard immunofluorescence microscopy.
